# Metabolomics of early blight (*Alternaria solani*) susceptible tomato (*Solanum lycopersicum*) unfolds key biomarker metabolites and involved metabolic pathways

**DOI:** 10.1038/s41598-023-48269-0

**Published:** 2023-11-29

**Authors:** Dhananjaya Pratap Singh, Sudarshan Maurya, Suresh Reddy Yerasu, Mansi Singh Bisen, Mohamed A. Farag, Ratna Prabha, Renu Shukla, Krishna Kumar Chaturvedi, Md. Samir Farooqi, Sudhir Srivastava, Anil Rai, Birinchi Kumar Sarma, Nagendra Rai, Tusar Kanti Behera

**Affiliations:** 1 ICAR-Indian Institute of Vegetable Research, Varanasi, 221305 India; 2 Pharmacognosy Department, College of Pharmacy, Cairo University, Cairo, Egypt; 3 ICAR-Indian Agricultural Statistics Research Institute, Library Avenue, New Delhi, India; 4 Indian Council of Agricultural Research, New Delhi, 110012 India; 5 Department of Mycology and Plant Pathology, Institute of Agricultural Sciences, Banaras Hindu University, Varanasi, 221005 India

**Keywords:** Biochemistry, Plant sciences

## Abstract

Tomato (*Solanum lycopersicum*) is among the most important commercial horticultural crops worldwide. The crop quality and production is largely hampered due to the fungal pathogen *Alternaria solani* causing necrotrophic foliage early blight disease. Crop plants usually respond to the biotic challenges with altered metabolic composition and physiological perturbations. We have deciphered altered metabolite composition, modulated metabolic pathways and identified metabolite biomarkers in *A. solani*-challenged susceptible tomato variety Kashi Aman using Liquid Chromatography-Mass Spectrometry (LC–MS) based metabolomics. Alteration in the metabolite feature composition of pathogen-challenged (*m/z* 9405) and non-challenged (*m/z* 9667) plant leaves including 8487 infection-exclusive and 8742 non-infection exclusive features was observed. Functional annotation revealed putatively annotated metabolites and pathway mapping indicated their enrichment in metabolic pathways, biosynthesis of secondary metabolites, ubiquinone and terpenoid-quinones, brassinosteroids, steroids, terpenoids, phenylpropanoids, carotenoids, oxy/sphingolipids and metabolism of biotin and porphyrin. PCA, multivariate PLS-DA and OPLS-DA analysis showed sample discrimination. Significantly up regulated 481 and down regulated 548 metabolite features were identified based on the fold change (threshold ≥ 2.0). OPLS-DA model based on variable importance in projection (VIP scores) and FC threshold (> 2.0) revealed 41 up regulated discriminant metabolite features annotated as sphingosine, fecosterol, melatonin, serotonin, glucose 6-phosphate, zeatin, dihydrozeatin and zeatin-β-d-glucoside. Similarly, 23 down regulated discriminant metabolites included histidinol, 4-aminobutyraldehyde, propanoate, tyramine and linalool. Melatonin and serotonin in the leaves were the two indoleamines being reported for the first time in tomato in response to the early blight pathogen. Receiver operating characteristic (ROC)-based biomarker analysis identified apigenin-7-glucoside, uridine, adenosyl-homocysteine, cGMP, tyrosine, pantothenic acid, riboflavin (as up regulated) and adenosine, homocyctine and azmaline (as down regulated) biomarkers. These results could aid in the development of metabolite-quantitative trait loci (mQTL). Furthermore, stress-induced biosynthetic pathways may be the potential targets for modifications through breeding programs or genetic engineering for improving crop performance in the fields.

## Introduction

Pathogenic interactions in plants initiate cellular metabolic reprogramming and by doing so, host plant counteracts with the damage caused due to the progression of infection^1^. In pathogenic invasion, a cascade of complex but integrated metabolic and molecular networks are triggered, leading to the real time activation of plant responses to influence sensing and cross talk between the interacting partners^2,3^. Progression of the disease activates different metabolic pathways in plants for the biosynthesis and accumulation of primary and secondary metabolites that either help them in instigating their survival ability by maintaining minimal growth or reducing pathogen invasion by organizing immune responses to combat the disease^4,5^. The biosynthesis and metabolism of chemically diversified plant-derived metabolites entwined with their diverse biological functions are typically linked with plant health, defense and survival in the environment^6,7^. Therefore, identifying chemical diversity, mapping the pathways with biological functions and understanding integrated responses at metabolic level in pathogen challenged plants can help prediction of the outcome of such interactions in system biology perspectives^8^. Such studies are important to pin point biological properties of multiscale network connections and significant biomarker metabolite signatures in plant pathogenic interactions^9^. Emerging metabolomics technologies entwined with bioinformatics tools and statistical modules are unequivocally becoming important to understand reprogramming of metabolites and their metabolic pathway in plants under plant-pathogenic interaction^10,11^. LC–MS is an indispensable tool not only for revealing biotic stress-linked significant biomarker metabolites that hold prospects of becoming mQTLs to assist stress breeding programs, but for exploring intrinsic cellular mechanisms that regulate plant responses in the environment also^12,13^.

Throughout the world, tomato is the second most cultivated crop globally with the production of 189 million tons from 5 mha of land as per FAOSTAT 2022^14^. Tomato, as a major horticultural crop for nutrition and human health is considered as model to decipher plant responses against abiotic and biotic stress challenges^15,16^. Worldwide, tomato is highly prone to > 200 diseases, probably because of the low genetic diversity and intensive selection during domestication and evolution^17^. Early blight disease caused by *A. solani* is a devastating necrotrophic disease of tomato causing extensive damage to foliar tissues^18^, due to which the crop usually suffers > 50% loses under field conditions^17^. Pathogenic interactions with susceptible plants impair growth and development by downsizing primary metabolism, even in the conditions where the pathogen could not cause disease or death of the plants^19^. Aside, consequent activation of bioenergetics in secondary metabolism in stressed plants also becomes high burden at the cost of growth and productivity^20^. Critical perturbations in the metabolism of susceptible plants post pathogen invasion drive key outcomes of the attempted infection^19^. This could be observed in substantial metabolic reprogramming in key biosynthetic pathways that allow plants to synthesize and accumulate diverse metabolic capabilities to overcome diseases^21^. Hence, uncovering metabolic responses in pathogen-challenged and non-challenged susceptible plants could reveal key changes in the pathways of defense, signaling, hormones and primary and secondary metabolites that determine pathogen progression^2^. It, therefore, became imperative to explore global metabolomic profiles of plant tissues to explore cellular metabolic reprogramming for ascertaining chemical readouts of plant defense^22^.

Although plant pathogen interactions were studied at biochemical and molecular levels^2^, our current understanding of susceptible plant responses against pathogens in terms of their intrinsic chemical diversity and metabolic pathway modulations is rather limited. We hypothesize that a comparative mapping of the global metabolome profile in susceptible variety of tomato grown under pathogen-challenged and non-challenged condition could reveal metabolomics response in plant tissues in unbiased manner. Accordingly, we compared the metabolome profiles between diseased and control plant leaf tissues and identified biomarkers that not only significantly discriminated the two contrasting samples but were also linked with key metabolic pathways which influenced plant growth, development and defense under pathogenic challenged conditions. Results from this study could pave the way for developing screening methods for resistance and susceptible crop varieties based on metabolite profiling, designing tools for disease diagnostics based on key biomarker metabolites and targeting mQTL development based on signature markers, defense-linked metabolic pathways and metabolite traits for their implications in stress breeding programs in the crop against biotic challenges.

## Results

**Figure 1:**
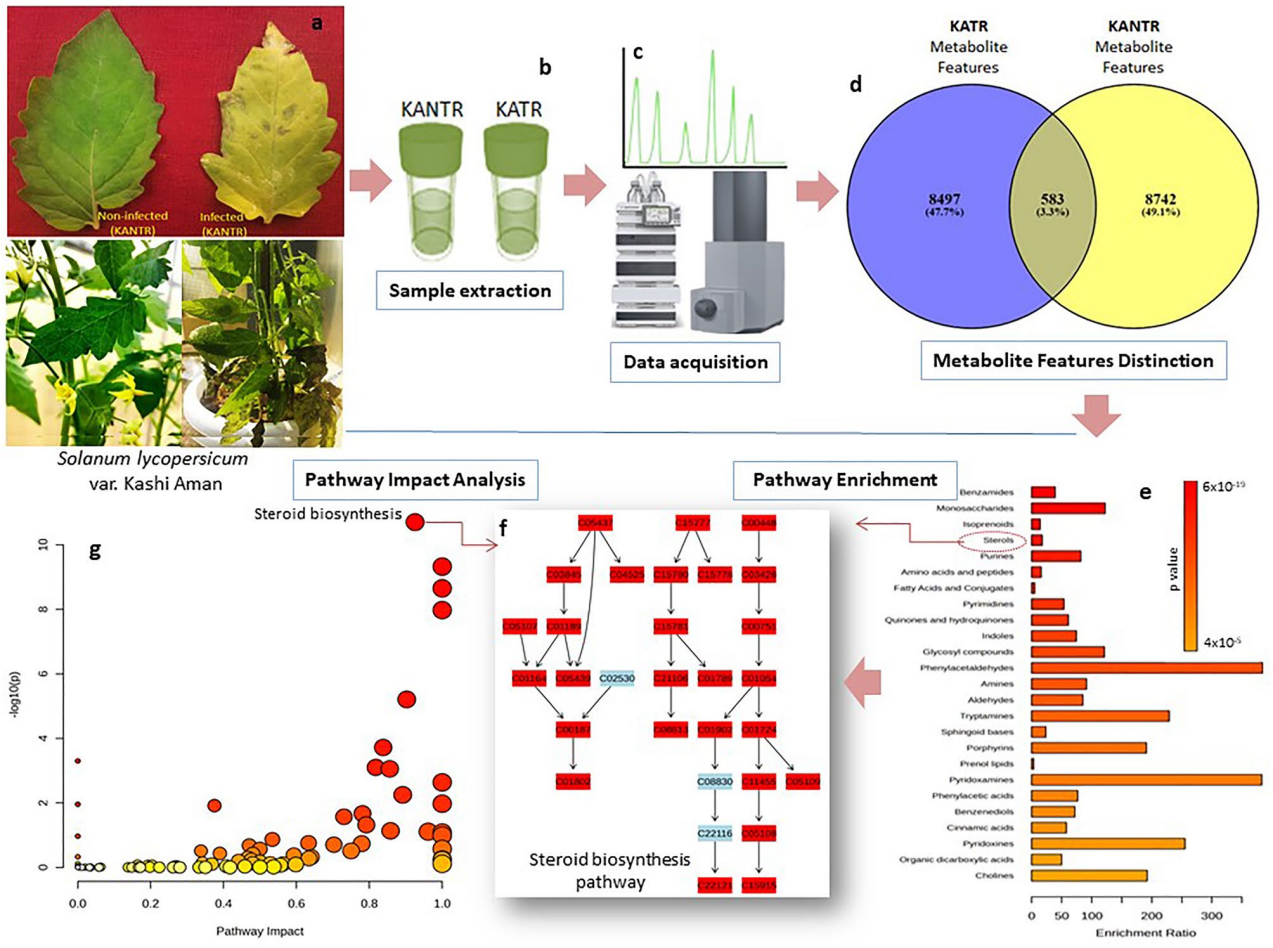
Representation of steps that highlight experimental study outline, (**a**) plant leaves challenged with *A. solani*, (**b**) metabolome extraction from pathogen-challenged and non-challenged leaves, (**c**) data acquisition using LC–MS, (**d**) feature exclusiveness analysis of metabolomics data sets in pathogen challenged and non-challenged susceptible tomato variety Kashi Aman, (**e**) pathway enrichment analysis, (**f**) steroid biosynthesis pathway (in KEGG) which have shown, (**g**) high impact in pathway impact analysis.

A comparative metabolite profiling of *A. solani* pathogen-challenged (KATR) and non-challenged (KANTR) tomato leaves was made to understand intrinsic metabolic changes. The disease incidence score of 67.6% indicated severity of the invasion in susceptible plants after 3 days of pathogen inoculation (Fig. 1a), following which the leaf tissues were extracted (Fig. 1b) and subjected to data acquisition (Fig. 1c) using LC–ESI–MS/MS spectra in negative ion mode that allowed better sensitivity in terms of peak intensity and numbers^23^. (Supplementary Figs S1 and S2). Metabolite compositional changes occurred between KATR and KANTR samples as shown by the feature exclusiveness in Venn diagram (Fig. 1d) which reflected 8487 (47.7%) infection-exclusive, 8742 (49.1%) non-infection exclusive and only 583 (3.3%) common metabolite features indicating metabolite variability. Functional annotation has resulted in metabolite classes, pathway enrichment and impacts (Fig. 1e,f,g). Furthermore, statistical and chemometric analyses employing defined parameters [*m/z*, retention time (*rt*), peak intensity and *p* values] elucidated signature biomarker metabolites having critical impacts against pathogenic invasion in the samples.

### Univariate and multivariate data analyses

**Figure 2:**
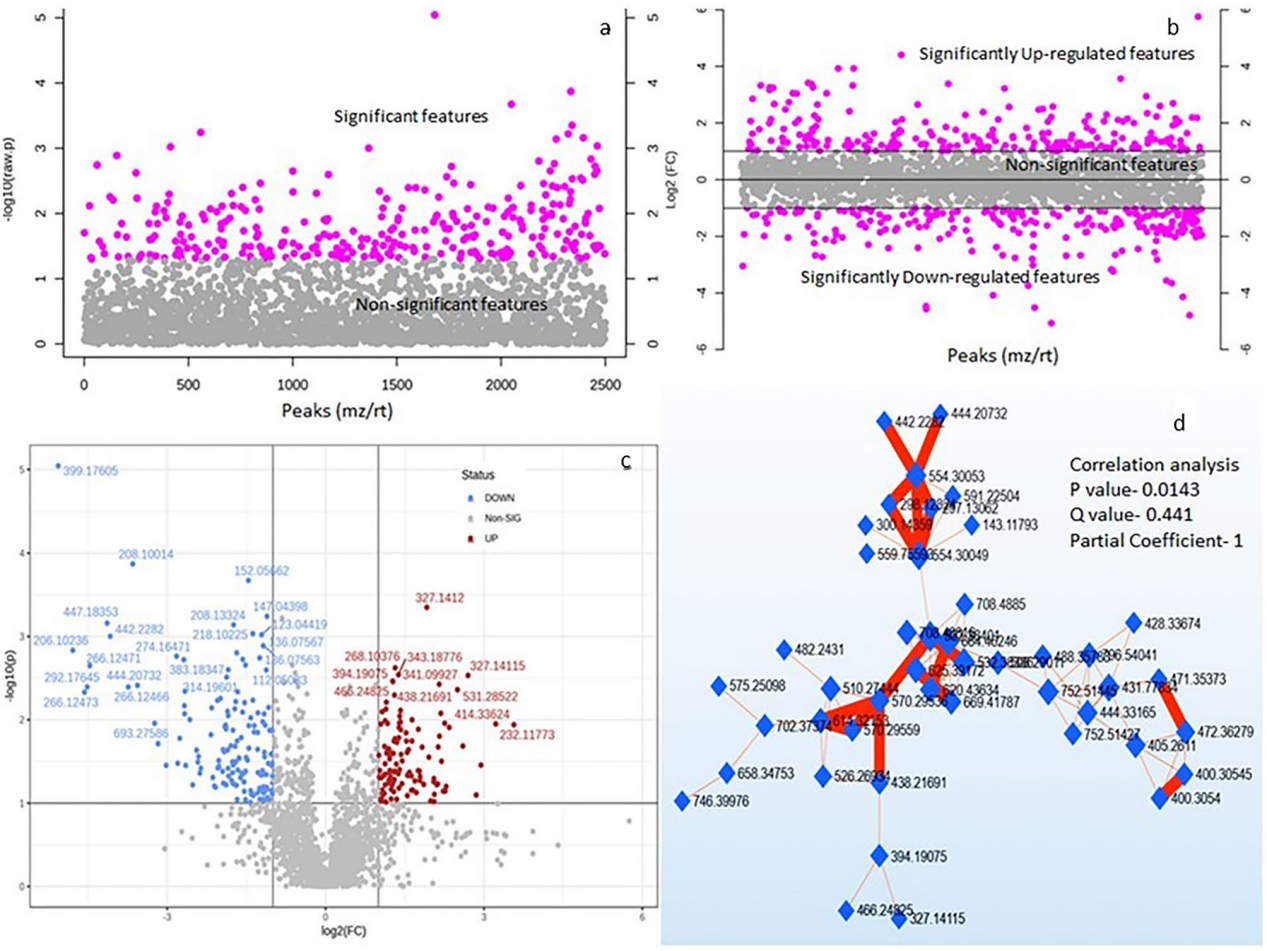
Univariate feature analysis in MataboAnalyst 5.0 (**a**) significance test in *t* test (Wilcoxon rank-sum tests) identified 661 significant metabolite features at (*p* ≤ 0.05); (**b**) fold change (FC threshold ≥ 2.0) analysis of metabolite features identified 481 significantly over- regulated and 548 down regulated metabolite features; (**c**) Volcano plot, up- and down regulated significant (*p* ≤ 0.05) metabolite features were clearly differentiated; (**d**) debiased sparse partial correlation (DSPC), partial correlations are established at *p* 0.0143, Q value 0.441 and partial coefficient 1.0 between those metabolites which usually go unidentified and remain unknown but represent bonafide entities for interpreting biological data.

**Figure 3:**
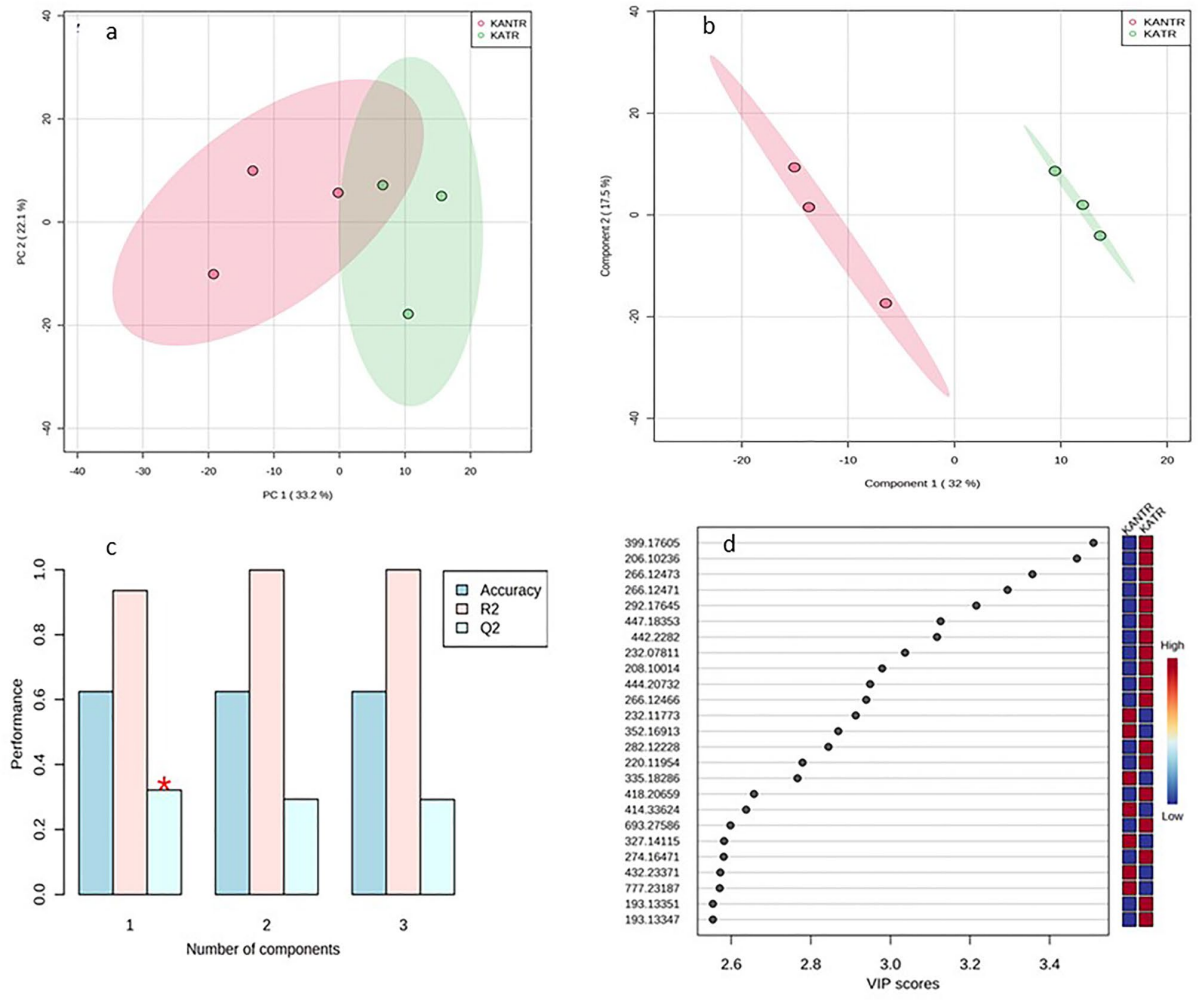
PCA and PLS-DA analysis; principal component analysis PCA score plot (**a**) showing distinct relationship between samples and differences in KATR versus KANTR samples along *x*-axis (PC1) and within groups along *y*-axis (PC2). PC1 and PC2 showed variance at 33.2% and 22.1% respectively at 95% confidence interval; partial least *squares*-*discriminant* analysis (PLS-DA) Scores plot of metabolite features (**b**) in PLS-DA model representing covariance between component 1 (32%) at *x* axis and component 2 (17.5%) at *y* axis; (**c**) cross validation of PLD-DA model with positive Q2 reflecting predictability and non-overftting of the model and top 25 annotated metabolites identified from metabolite features with variable importance at *x* axis. A higher VIP score (> 1.0) for metabolites represent higher importance in influencing the scores most.

Univariate analysis showed 616 significant metabolite features in *t*-test at *p* ≤ 0.05 (Fig. 2a). Fold change (FC** ≥ **2.0) analysis suggested 481 significantly up- and 548 down regulated metabolite features (Fig. 2b) which were clearly differentiated in a Volcano plot in KATR-KANTR samples (Fig. 2c). With the help of DSPC, partial correlations were established between the unknown metabolite features that were supposed to become bonafide compounds for interpreting biological data (Fig. 2d). Unsupervised multivariate Principal Component Analysis (PCA) model descriptively highlighted trends of relationships between and within KATR-KANTR samples for distribution in the two principal components, PC1 and PC2 and pathogen-challenged vs non-challenged clustering. PCA-score plot revealed variance of 33.2% along PC1 and 22.1% along PC2 to indicate differentiation of metabolite features (Fig. 3a). A more comprehensive and conclusive interpretation of the metabolic responses in pathogen-challenged plant was obtained from supervised PLS-DA and orthogonal PLS-DA (OPLS-DA) models, which helped to extract multiple metabolite variables that govern discrimination between the samples lead to biomarker identification^24,25^. In PLS-DA, metabolite features showed discrimination between the KATR and KANTR having covariance of 32% at component 1 (*x*-axis) and 17.5% at component 2 (*y*-axis) (Fig. 3b). Cross validation analysis of PLD-DA model showed positive Q2 reflecting predictability and non-overftting of the model (Fig. 3c). The variable importance in projection (VIP) scores of top 25 metabolite features (VIP scores ≥ 1) indicated their major contribution in sample segregation (Fig. 3d).

**Figure 4:**
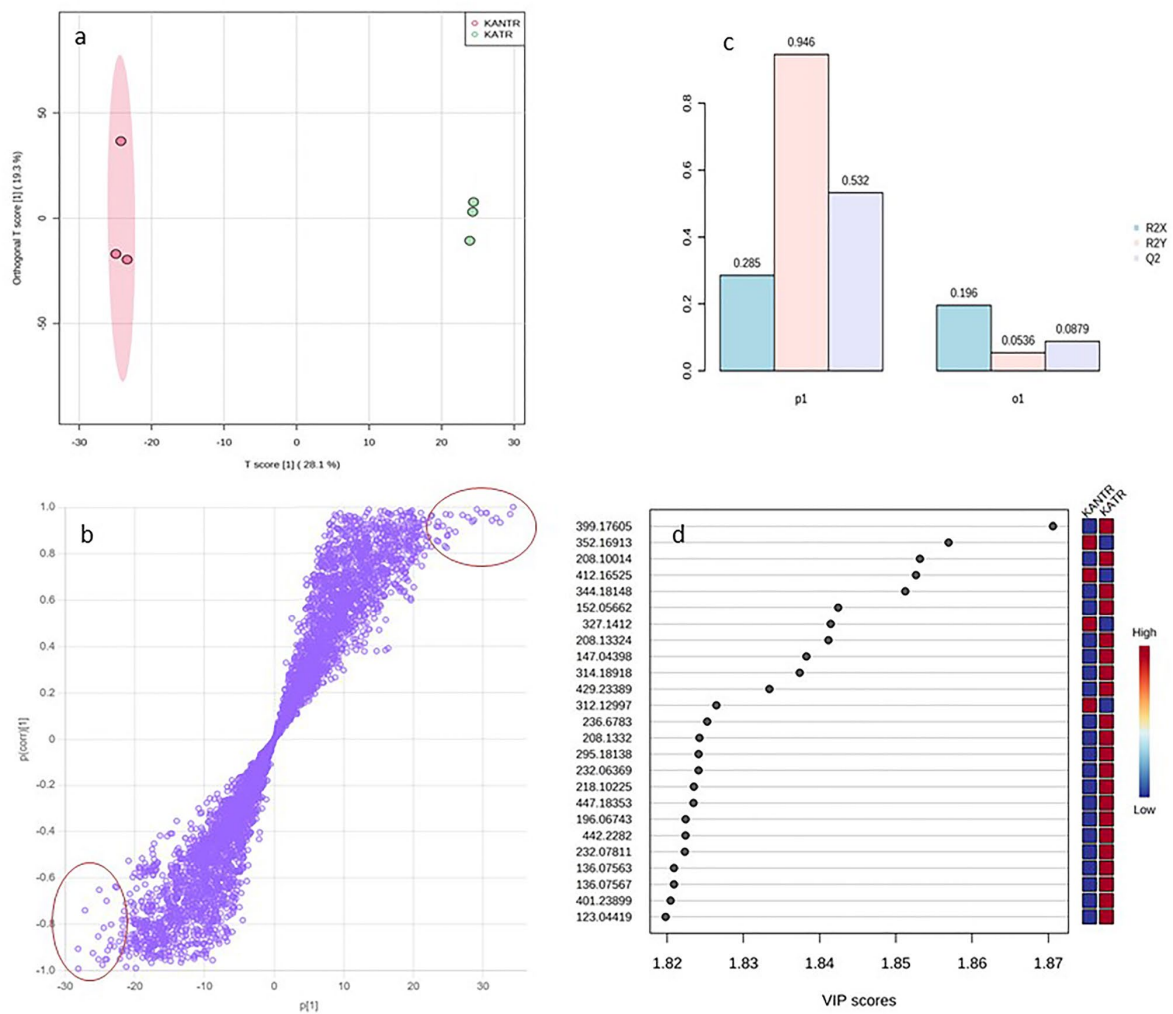
Orthogonal partial least square-discriminant analysis (OPLS-DA) for metabolite features in KATR-KANTR samples. (**a**) Score plot represented sample discrimination; S-plot (important features) facilitated visualization of the variable influence of metabolite features combining the covariance and correlation loading profile of the metabolite features in KATR-KANTR samples. The plot identifies putative biomarker metabolite features as negative outliers at bottom left and positive outliers at top right (circled in red) having high influence on the discrimination of the groups (**b**); cross validation of the model with positive Q2 value suggesting a predictive and non-over-fitted model (**c**) and VIP plot (*x* axis) identifying 25 top most putatively identified discriminant metabolites with VIP > 1.0 having higher impact on discrimination between sample groups.

OPLS-DA model further added explanatory and interpretational benefits over PLS-DA^24^. OPLS-DA Score-plot, in which the sample discrimination was represented by T score [1] 28.1% and orthrogonal T score [1] 19.3% (Fig. 4a). The reliability of the analysis was presented in S-plot by *p*(corr) (*x* axis) having a value between + 1 to −1 showing features of unique *m/z* and *rt* with high covariance to depict discriminatory biomarker features (Fig. 4b). Positive discrimination contributing features remained as outliers at the top right quadrant while those appeared at the lower left contributed negatively in response to the plants exposed to pathogenic challenge. Cross validation of the model suggested positive Q2 values showing high predictability and non-overftting (Fig. 4c). Metabolite discriminant variables contributing to OPLS-DA model were ranked with the VIP scores and based on the high VIP score (≥ 1.0), top 25 metabolite variables strongly contributing to the variation were *m/z* 399.17605, 352.16913, 208.10014, 412.16525, 344.18148, 151.05662, 327.1412, 208.13324, 147.04398, 314.18918 etc. (Fig. 4d). Most of these metabolite features were found up- regulated in KATR.

### Up and down regulated discriminant annotated metabolites

KEGG identifier of the metabolite features was obtained from functional annotation using MetaboAnalyst 5.0 and further validatation was performed with PLANT CYC PMN database of *S. lycopersicum.* FC analysis indicated 481 significantly up- and 548 down regulated metabolite features at a threshold of ≥ 2.0. The features when clubbed with the VIP score of ≥ 1.0 in OPLS-DA model led to putative identification of 43 up- and 23 down- regulated metabolite discriminants as tabulated with their *m/z*, FC, VIP score, *rt*, KEGG id, putative name and metabolite class. Melatonin (*m/z* 232.117) was the key annotated metabolite with maximum FC (11.84) followed by glucose 6-phosphate (*m/z* 405.261; FC 8.26), sphingosine (*m/z* 358.505; FC 7.22),zeatin-7-β-d-glucoside (*m/z* 396.222; FC 6.06) and serotonin (*m/z* 175.111; FC 3.21) (Table 1). In contrast, down regulated metabolites included histidinol (*m/z* 122.096; FC 0.42), 4-aminobutyraldehyde (*m/z* 132.060; FC 0.31), propanoate (*m/z* 133.060; FC 0.27), tyramine (*m/z* 182,081; FC 0.44) and linalool (*m/z* 191.106; FC 0.41) along with other metabolites (Table 2).

**Table 1:** Up regulated putatively annotated metabolites* based on significant FC (≥ 2.0) (*p* ≤ 0.05) and VIP score (≥ 1.0) in *A. solani* pathogen-challenged *S. lycopersicum* susceptible var. Kashi Aman.

S. no.	*m/z*	r.t. (min)	FC value	VIP score (OPLS_DA model)	KEGG identifier	Annotation	Metabolite class
1.	358.505	7.68	7.22	1.42	C00319	Sphingosine	Lipids
2.	398.241	10.46	5.32	1.21	C04525	Fecosterol	Lipids
3.	227.127	6.10	4.80	1.46	C16311	12-Oxo-9-dodecenoic acid	Lipids
4.	459.350	13.27	4.17	1.43	C15796	22-Hydroxycampest-4-en-3-one	Lipids
5.	311.221	12.23	4.00	1.22	C14827	Hydroperoxyoctadeca-10,12-dienoic acid	Lipids
6.	531.285	7.10	3.1	1.67	C03313	Phylloquinol	Lipids
7.	398.24	10.74	3.01	1.55	C15777	Episterol	Lipids
8.	527.266	7.58	2.97	1.32	C15793	Typhasterol	Lipids
9.	244.191	8.49	2.76	1.11	C09684	Humulene	Lipids
10.	319.205	4.92	2.53	1.18	C19616	18-Hydroxyoleic acid	Lipids
11.	401.141	4.59	2.4	1.28	C06095	Gibberellin A44 open lactone	Lipids
12.	273.184	9.11	2.16	1.19	C01226	12-Oxophyto-10,15-dienoate	Lipids
13.	257.138	5.61	2.02	1.51	C16311	12-Oxo-9-dodecenoic acid	Lipids
14.	289.068	1.47	2.64	1.58	C05401	Galactosylglycerol	Carboydrateate
15.	171.026	1.52	2.29	1.49	C02637	3-Dehydroshikimate	Carboxylate
16.	217.034	2.15	2.25	1.71	C02631	2-Isopropylmaleate	Carboxylate
17.	148.038	1.09	3.23	1.28	C00450	Tetrahydropyridine-2-carboxylate	Carboxylate
18.	335.221	14.06	3.11	1.73	C19620	9,10-Epoxy-18-hydroxystearate	Lipids
19.	229.107	5.52	2.39	1.37	C01909	Dethiobiotin	Lipids
20.	163.075	6.06	2.91	1.65	C06001	3-Hydroxyisobutyrate	Carboxylate
21.	229.154	0.83	2.93	1.54	C17621	8-Hydroxygeraniol	Terpenoid
22.	301.215	12.5	5.07	1.71	C11874	Kaurenic acid	Terpenoid
23.	405.261	12.72	8.26	1.00	C00092	Glucose 6-phosphate	Carbohydrate
24.	396.222	8.25	6.06	1.67	C16443	Zeatin-7-β-d-glucoside	Carbohydrate
25.	201.036	1.528	2.59	1.54	C01019	Fucopyranose	Carbohydrate
26.	232.117	2.98	11.84	1.71	C01598	Melatonin	Nitrogenous
27.	531.285	7.12	5.64	1.78	C15802	6-Deoxocastasterone	Sterols
28.	271.114	11.84	2.46	1.55	C00931	Porphobilinogen	Porphyrin
29.	515.006	0.81	4.53	1.73	C18064	Pyropheophorbide *a*	Porphyrin derivative
30.	341.099	8.98	4.44	1.79	C10646	Lariciresinol	Polyphenols
31.	242.283	12.31	4.38	1.64	C02029	Dihydrozeatin	Nitrogenous
32.	228.120	10.89	4.13	1.5	C00371	Zeatin	Nitrogenous
33.	356.279	12.69	4.06	1.08	C01137	Adenosylmethionin-amine	Peptides
34.	175.111	7.61	3.21	1.27	C00780	Serotonin	Nitrogenous
35.	279.098	11.51	3.13	1.25	C00643	5-Hydroxy-l-tryptophan	Amino acid
36.	222.083	4.90	2.68	1.71	C00140	*N*-Acetyl-d-glucosamine	Amino sugar
37.	259.021	1.74	3.28	1.45	C00352	Glucosamine 6-phosphate	Amino monosaccharide
38.	313.104	8.56	2.75	1.18	C00921	Dihydropteroate	Folate derivative
39.	243.047	0.79	2.72	1.48	C00307	CDP-choline	Choline
40.	289.068	1.35	2.41	1.51	C05401	Uridine	Nitrogenous
41.	339.177	11.27	2.00	1.28	C20693	Carlactone	Tetrahydropyridine

*r.t.* retention time, *FC* fold change.

*Annotated using PLANT CYC PMN (*Solanum lycopersicum* database) and KEGG compound search.

**Table 2:** Down regulated putatively annotated metabolites* based on significant FC (≥ 2.0) (*p* ≤ 0.05) and VIP score (≥ 1.0) in *A. solani* pathogen challenged susceptible tomato leaves.

S. no.	*m/z*	r.t.(min)	FC value	VIP Score (OPLS-DA model)	KEGG identifier	Annotation	Metabolite class
1.	122.096	2.19	0.42	1.23	C00860	Histidinol	Nitrogenous
2.	132.060	1.31	0.31	1.57	C00555	Aminobutyraldehyde	Nitrogenous
3.	133.060	0.88	0.27	1.72	C00163	Propanoate	Carboxylic acid
4.	145.047	0.89	0.45	1.22	C00966	2-Dehydropantoate	Carboxylic acid
5.	145.085	4.35	0.49	1.44	C08492	3-Hexenol	Alcohol
6.	147.044	1.26	0.46	1.85	C00576	Betaine aldehyde	Nitrogenous
7.	152.056	1.09	0.36	1.86	C03912	1-Pyrroline-5-carboxylate	Cyclic imino acid
8.	173.117	9.76	0.46	1.22	C00568	4-Aminobenzoate	Nitrogenous
9.	163.075	6.80	0.46	1.37	C06001	3-Hydroxyisobutyrate	Carboxylic acid
10.	180.136	1.33	0.41	1.68	C05332	2-Phenylethylamine	Nitrogenous
11.	182.081	0.90	0.44	1.46	C00483	Tyramine	Amino acid
12.	188.070	4.08	0.49	1.53	C01879	Pyroglutamic acid	Amino acid
13.	157.122	5.38	0.15	1.09	C11863	Gibberellin A9	Terpene
14.	191.106	8.60	0.41	1.74	C11389	Linalool	Terpene
15.	184.057	0.80	0.47	1.30	C02273	Digalacturonic acid	Carbohydrate
16.	185.032	0.80	0.32	1.61	C06007	2,3-Dihydroxy-3-methylpentanoate	Lipids
17.	201.112	8.87	0.36	1.14	C19757	Hex-3-en-1-yl acetate	Lipids
18.	277.215	9.78	0.49	1.05	C00249	Palmitic acid	Lipids
19.	295.226	9.77	0.42	1.31	C01595	Linoleic acid	Lipids
20.	189.015	0.84	0.31	1.58	C00379	Xylitol	Carbohydrate
21.	203.106	12.51	0.49	1.51	C04083	Isopentenyladenine	Naturally occurring cytokinin
22.	218.102	5.42	0.38	1.84	C00978	*N*-Acetylserotonin	Nitrogenous
23.	257.113	1.55	0.49	1.29	C00449	Saccharopine	Alpha aminoadipate

*r.t.* retention time, *FC* fold change.

*Annotated using PLANT CYC PMN (*Solanum lycopersicum* database) and KEGG compound search.

### Biomarkers identification for *A. solani* infection

**Figure 5:**
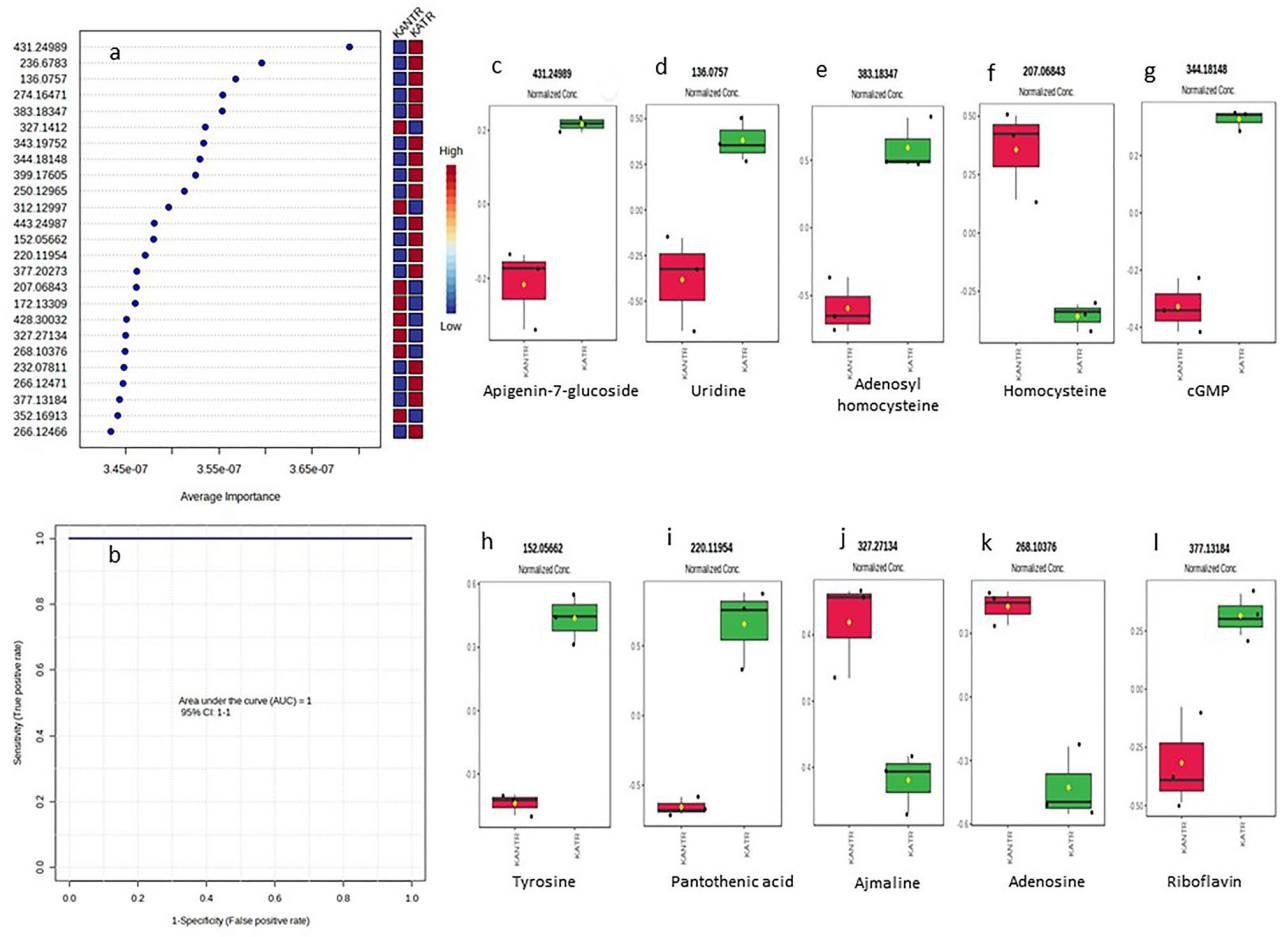
Multivariate exploratory ROC curve analysis showing 25 potential metabolite biomarkers selected by applying support vector machine (SVM), partial least squares discriminant analysis (PLS-DA), and random forests (**a**); AUC curve with 1 value showing high predictability of the model at 100 features (model 6) in MetaboAnalyst5.0 (**b**); and annotated up regulated metabolite biomarkers apigenin-7-glucoside (**c**), uridine (**d**), adenosyl homocysteine (**e**), cyclic guanosine monophosphate (cGMP) (**g**), tyrosine (**h**), pantothenic acid (**i**), riboflavin (**l**) and down regulated metabolites homocysteine (**f**), ajmaline (**j**) and adenosine (**k**).

Multivariate ROC curve based exploratory analysis, that leads to automated identification of important features and performance evaluation, has led to identify top 25 signature biomarker metabolite features on the parameters of PLS-DA algorithm and average importance measure (Fig. 5a). The area under the curve (AUC) was found to be 1 at 95% confidence band upon selection of the model with 100 features, meaning that the predictability of the model was high and the classifier metabolites perdected distinctly high sensitivity and specificity (Fig. 5b). Biomarker feature annotation using MassBank and PMN *S. lycopersicum* database has led to identify apigenin-7-glucoside (*m/z* 431.24989), uridine (*m/z* 236.6783), homocyctine (*m/z* 207.6843), adenosyl-homocysteine (*m/z* 383.18347), guanosine cyclic monophosphate (cGMP) (*m/z* 344.18148), tyrosine (*m/z* 152.05662), pantothenic acid (*m/z* 220.11954), adenosine (*m/z* 268.10376), adenosine diphosphate (*m/z* 428.30032), azmaline (*m/z* 327.27134) and riboflavin (*m/z* 377.13184) out of 25 top selected features. Among these biomarker metabolites, apigenin-7-O-glucoside, uridine, adenosyl homocysteine, cGMP, tyrosine, pantothenic acid and riboflavin were up-regulated (Fig. 5c,d,e,g,h,i,l) )while homocysteine, ajmaline and adenosine (Fig. 5f,j,k) were down regulated in disease challenged plants (Fig. 5c–l).

### Metabolic pathway enrichment and impact analyses

**Figure 6:**
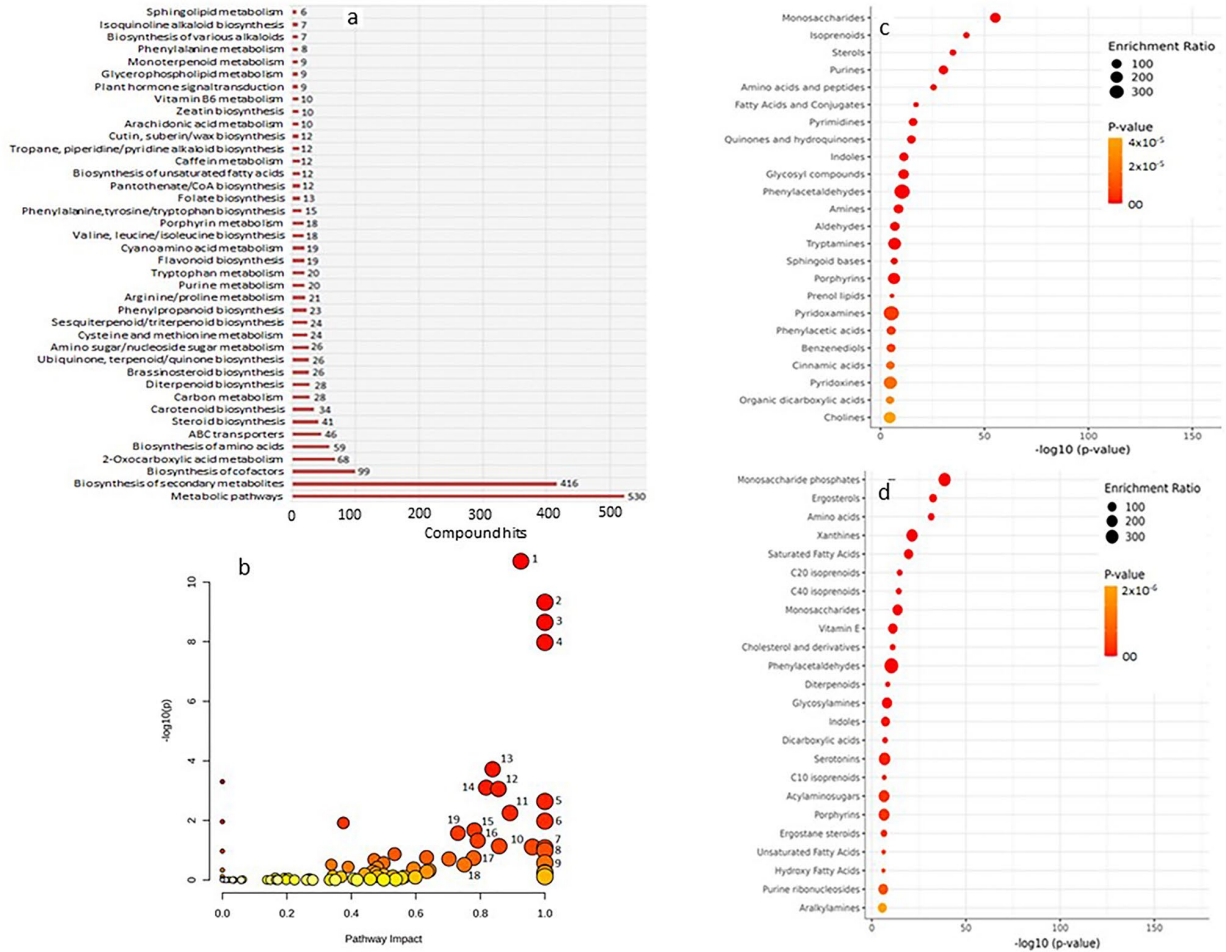
Pathway enrichment analysis using KEGG Mapper mapped metabolite feature hits (number in parenthesis) with different biosynthesis and metabolic pathways (**a**) (minimuml compound hit values from 1 to 5 are not shown); Pathway impact analysis showing impact of significantly different pathways (*p* ≤ 0.05) influencing metabolic responses in plant leaves (**b**); Size of the circle indicates the impact of the pathway while the Color from red to yellow represents the high to low significance levels; Identified pathways were (1) steroid biosynthesis, (2) diterpenoid biosynthesis, (3) brassinosteroid biosynthesis, (4) sesquiterpenoid & triperpenoid biosynthesis, (5) one carbon pool by folate, (6) biosynthesis of secondary metabolites, (7) isoquinoline biosynthesis, (8) betainn biosynthesis, (9) linoleic acid metabolism, (10) vitamin 6 metabolism, (11) ubiquinone & other terpenoid biosynthesis, (12) pentose phosphate pathway, (13) carotenoid biosynthesis, (14) valine, leucine & isoleucine biosynthesis, (15) pentose & glucoronate interconversions, (16) tryptophan metabolism, (17). α-linoleic acid metabolism, (18) cutin, suberine & wax biosynthesis, (19) arginine & proline metabolism; Enrichment analysis of up-regulated 481 (**c**) and down regulated 548 (**d**) metabolite features obtained from FC threshold ≥ 2.0 into different compound groups using MetaboAnalyst 5.0.

Annotated metabolites having KEGG identifiers were subjected to KEGG Mapper for pathway mapping to obtain their enrichment in metabolic pathways. The mapping suggested metabolites hits in 106 metabolic pathways of *S. lycopersicum* (*sly*) database. Two major pathways having maximum metabolite hits were ‘metabolic pathways’ (n = 530) and ‘biosynthesis of secondary metabolites’ (n = 416) (Fig. 6a). Besides, significant number of hits were also related to the biosynthesis of cofactors (n = 99), oxocarboylic acids (n = 68), amino acid metabolism (n = 59), steroids (n = 41), carotenoids (n = 34), brassinosteroids (n = 26) and ubiquinone/terpenoid-quinone biosynthesis (n = 26) along with other pathways with low numbers (Fig. 6a). Pathway impact (PI) was assessed the impact of different pathways on the response of the plants against pathogen attack (Fig. 6b). The analysis combines the centrality and pathway enrichment results and adds up the important measures for the high impact pathways. The most important highly enriched pathways having significantly (*p* ≤ 0.05) high impact in pathogen challenged tomato plants included those involved in the biosynthesis of diterpenoids (PI 1.0001), brassinosteroids (PI 1.0), sesquiterpenoid and triperpenoids (PI 1.0), steroids (PI 0.925), secondary metabolites (PI 1.0), carotenoids (PI 0.837), ubiquinone/other terpenoids (PI 0.891), valine, leucine/isoleucine (PI 0.817) and monoterpenoids (PI 0.375) (Fig. 6b, Table 3). Apart from secondary metabolic pathways, others included metabolism of tryptophan (PI 0.791), arginine/proline (PI 0.730), one carbon pool by folate (PI 1.0) and pentose and glucuronate interconversions (PI 0.781) (Table 3).

**Table 3:** Pathways having significantly (*p* < 0.05) high impact in pathogen challenged tomato leaves.

Sr. no.	Pathway name	Match status*	*p* value	Pathway impact
1	Diterpenoid biosynthesis	28/28	4.71 × 10^–10^	1.0001
2	Brassinosteroid biosynthesis	26/26	2.23 × 10^–9^	1.0
3	Sesquiterpenoid and triterpenoid biosynthesis	24/24	1.05 × 10^–8^	1.0
4	One carbon pool by folate	8/8	0.0023	1.0
5	Steroid biosynthesis	42/45	1.98 × 10^–11^	0.925
6	Ubiquinone and other terpenoid-quinone biosynthesis	26/38	0.0056	0.891
7	Pentose phosphate pathway	16/19	8.81 × 10^–4^	0.856
8	Carotenoid biosynthesis	32/43	1.91 × 10^–4^	0.837
9	Valine, leucine and isoleucine biosynthesis	18/22	7.95 × 10^–4^	0.817
10	Tryptophan metabolism	18/28	0.047	0.791
11	Pentose and glucuronate interconversions	12/16	0.021	0.781
12	Arginine and proline metabolism	22/34	0.026	0.730
13	Monoterpenoid biosynthesis	8/9	0.012	0.375

*Matching status of the compounds in the data sets with those in the KEGG Compound database of *Solanum lycopersicum* {*sly*}.

Distribution of annotated metabolites across different chemical classes (464 main chemical class metabolite sets and 1072 sub chemical class metabolite sets in MetaboAnalyst 5.0) was grouped with significantly high enrichment to include isoprenoids (n = 52), fatty acids and conjugates (n = 42), sterols (n = 39), amino acids and peptides (n = 30), monosaccharides (n = 31), prenol lipids (n = 21), quinols and hydroquinones (10), cholines (n = 2), phenylpropanoids and phenols (n = 5), sphingonoid bases (n = 6) and phenylacetaldehydes (n = 3) all showing statistically significant *p* values (*p* ≤ 0.05) (Fig. 6c). Enrichment with sub class metabolite sets grouped the hits into ergosterols (n = 17), amino acids (n = 26), isoprenoids (C20 & C40) (n = 29), xanthines (n = 10), cholines and cholesterol derivatives (n = 15), serotonin (n = 4), diterpenoids (n = 10) and dicarboxylic acids (n = 7) along with other chemical classes (Fig. 6d). Metabolomic analysis also suggested some overlappings in the metabolite data sets which makes the metabolome data more representative, interpretable and understandable.

### Pathway collage and metabolite network analysis

**Figure 7:**
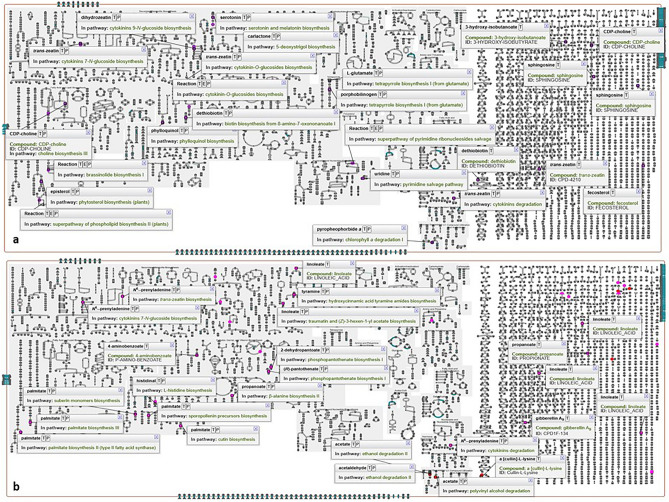
Pathway collage diagram showing specified set of pathways in PMN using *Solanum lycopersicum* organism database. Up- (**a**) and down- (**b**) regulated discriminating annotated metabolites based on FC threshold ≥ 2.0 and VIP > 1.0 were shown along with their involvement in metabolic pathways in the ‘cellular overview’ of the pathway collage diagram.

**Figure 8:**
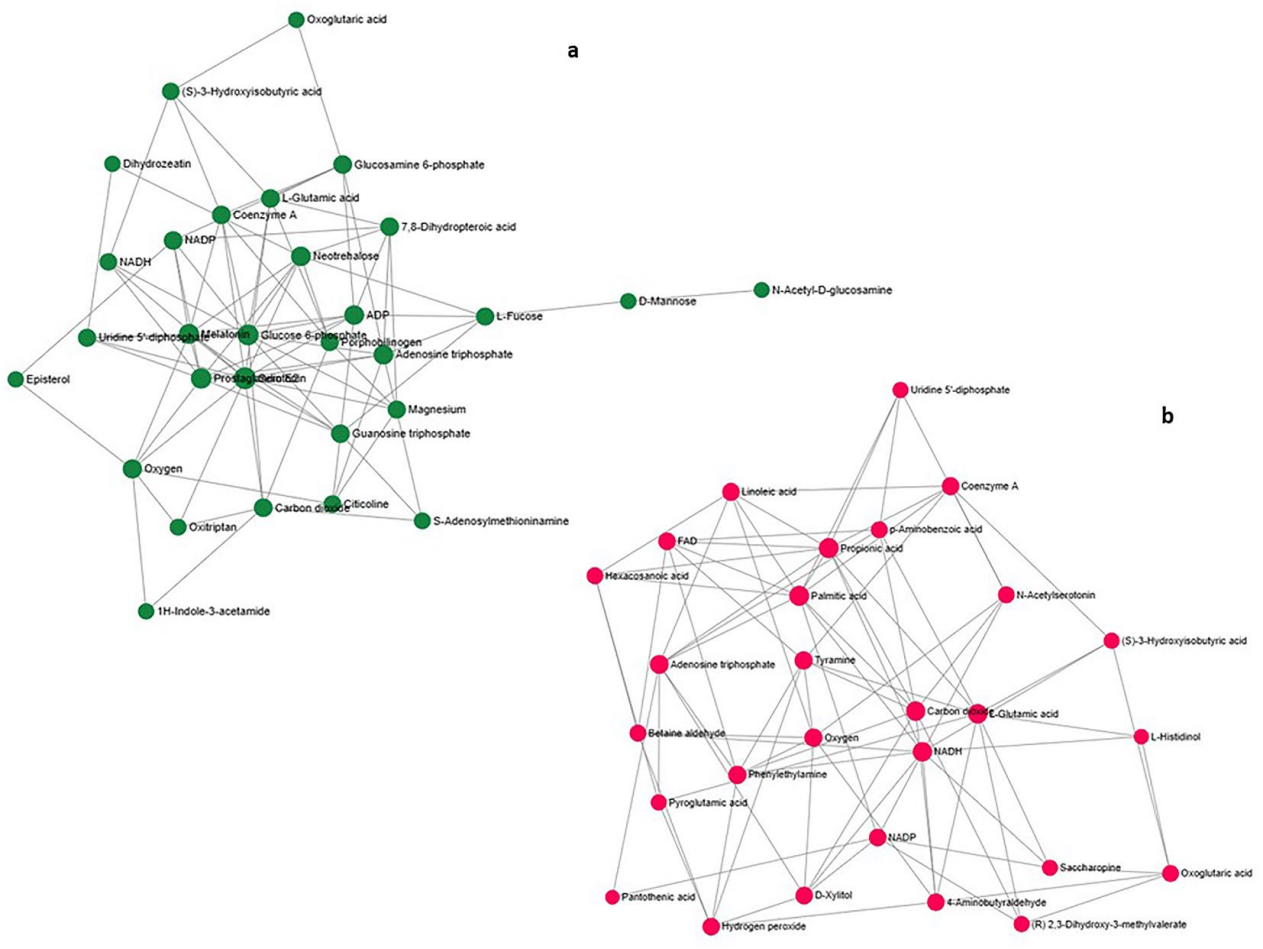
Network interaction analysis. Metabolite-metabolite interaction network for over regulated (nodes 30, edges 83, seeds 16) (**a**) and down regulated (nodes 28, edges 84, seeds 16) (**b**) annotated metabolites identified on the basis of FC threshold ≥ 2.0 (*p* < 0.05) and VIP (≥ 1.0). Colored nodes (green for over- regulated and red for down regulated) represent metabolites having interactive networks with biologically and functionally related other metabolites.

Pathway Collage diagram for up- and down- regulated putatively identified metabolites in PMN using *S. lycopersicum* database was shown to the relatedness of metabolites with specified set of pathways. The ‘cellular overview’ of the pathway collage diagram uniquely helped in showing specific pathways of the up and down regulated annotated metabolites (Fig. 7a,b). Further, a metabolic network showing metabolite relationship patterns among the putatively annotated metabolite signatures was prepared. The metabolite-metabolite interaction network (MetaboAnalyst 5.0), highlighted a potential functional relationship between annotated metabolites. The network displayed how metabolites are interconnected with their respective biosynthetic and metabolism pathways. Up- regulated discriminatory metabolites (FC ≥ 2.0; VIP ≥ 1.0) were represented by 30 nodes, 83 edges, and 16 seeds (Fig. 8a). Likewise, the down regulated metabolite interactions in the network were represented by 28 nodes, 84 edges and 16 seeds (Fig. 8b). Nodes of the representative up-regulated metabolites (green) were shown to be connected with oxoglutaric acid, glutamic acid, coenzyme A, dihydrozeatin, episterol, mannose, fucose, dihydropteroic acid, glucosamine-6-phosphate, etc. In network of down regulated metabolites, nodes of representatibe metabolites (red) showed connections with linoleinic acid, propionic acid, betaine aldehyde, pyroglutamic acid, pantothenic acid, tyramine, oxoglutaric acid, histidinol, aminobenzoic acid and acetylserotonin.

## Discussion

We examined metabolome of a less explored plant pathogen system of susceptible tomato-*A. solani* to assess complex metabolic changes in metabolic composition, key biosynthetic pathways and metabolite biomarkers. Comprehensive metabolic changes, as were observed, could help in identifying plant responses against stresses and early detection of pathogen infection^28^. Statistical analysis revealed 616 significant metabolite features (*t*-test, *p* ≤ 0.05) and revealed significantly up (481) and down (548) regulated metabolite features at FC threshold (**≥ **2.0). Functional analysis further linked enriched metabolite features with diverse metabolic pathways and putative annotation of metabolites was also obtained. The diversity in metabolite features and their differential regulation reflected dynamic and influential metabolic reprogramming in leaf tissues post pathogen inoculation, as has been observed in other plant-pathogen systems^26^.

PLS-DA and OPLS-DA results not only showed clear discrimination between the groups, but also showed predictive ability to reveal critical importance of potentially annotated differentiating discriminant metabolites with VIP score > 1.0 exerting high discriminatory power (Figs. 3, 4). These models have been effectively employed for discrimination purposes^24^ after proper cross validation based on R^2^Y and Q^2^ values, where positive Q2 signifies non-overfitting25. Cross model validation in metabolomics data has largely been advocated in the experiments with low numbers of samples with large variables for the accurate inference of class differentiation^27^. Top 25 metabolite features having VIP score > 1.0 from both the models were believed to be the predominantly abundant discriminant metabolite features supposed to be involved in prominent biological role against biotic stress in tomato plants.

Among the up-regulated 41 discriminant metabolite signatures, lipids were the major class followed by carboxylic acids, amino acids and fatty acids. These class of metabolites are supposed to mediate signaling and modulate effector-triggered systemic immunity in plants^29,30^. Terpenoids, steroids, alkaloids, porphyrins, polyphenols, folate derivatives and indoles were typical differential secondary metabolic groups accumulated in plants as an impact of disease challenge. Melatonin and serotonin derived from catalysis of amino acids in tomato are reported to regulate plant growth under stress^31^. These two indoleamines are being reported for the first time in tomato in response to early blight (*A. solani*). They possess antioxidative and growth-inducing properties and provide beneficial opportunities for stress acclimatization^32^. Porphyrins and other metabolites pyropheophorbide *a* and porphobilinogen are the precursors of chlorophyll and vitamins that regulate stress-responsive genes and ROS-mediated complex metabolic networks^33^. Other antioxidants showing differential response included vitamins (K, E, biotin), are potentially known to minimize ROS induced cell damage under biotic stress^34^.

Majority of the nitrogenous metabolites including tyramine, histidinol, phenylethylamine, betaine aldehyde, pyroglutamic acid and N-acetylserotonin were down regulated in pathogen-challenged plants. Plant responses to biotic and abiotic stressors are controlled by the interaction between nitrogen (N) and polyamines (PA), which is connected to secondary metabolic pathways^35^. Metabolites like palmitic acid, linoleic acid, 2,3-Dihydroxy-3-methylpentanoate and others including pantothenate and gibberellin A9 which are known antioxidants, metabolic signals, mediator of biosynthetic pathways, antimicrobials, resistance inducers and are structural cellular components^36–38^ were also down regulated. A shift from primary to secondary metabolism and compromise in production of primary metabolites over secondary metabolites under stress conditions has been observed in plants^39^. Also, for a favorable energy balance required for defense, up regulation of defense-linked metabolic pathways seems compensated by the down regulation of genes for other pathways^19^. In such circumstances, down regulated accumulation of secondary metabolites in susceptible tomato plants may be considered as become pathogen-mediated cellular response of susceptibility that may influence disease progression in plants^40^.

Putatively annotated biomarkers in multivariate ROC curve analysis included apigenin-7-glucoside, uridine, homocyctine, adenosyl-homocysteine, cGMP, tyrosine, pantothenic acid, adenosine, riboflavin and azmaline (Fig. 5a). ROC is widely used to predict diagnostic biomarkers with high confidence^41^ based on the potential discriminant performance of metabolite features^42^ as shown by the AUC being 1 (Fig. 5b). Up regulation of majority of putative biomarker metabolites suggested strong evidence of their dominant role in defense in disease challenged plants. Enhanced synthesis and accumulation of flavonoids and flavones was shown to be a prominent feature of altered metabolome^43^. This was supported by the evidence of enrichment of flavonoid biosynthesis in our results (Fig. 6a). Up regulated accumulation of biomarker metabolite apigenin-7-glucoside in disease challenged plant leaves in the results reflects its prominent defense role. Presence of uridine, a pyrimidine nucleoside neurotransmitter primarily found in tomato^44^ has been reported for increasing stress tolerance in plants^45^ and its enhanced accumulation as biomarker is supposed to have defense role in challenged plants. Likewise, methionine precursor non-proteinogenic amino acid homocysteine (Hcy) was shown to represent metabolic disorder in plants under challenged conditions^46^. Enrichment of cysteine and methionine metabolism has been reflected by our results (Fig. 6a). Hcy synthesized form redox-sensitive methionine metabolism, is essential for the formation of ethylene and polyamines which have crucial in plant defense^47^. Accumulation of S-adenosylhomocysteine that undergoes conversion into Hcy by a reaction catalyzed by S-adenosylhomocysteine hydrolase (SAHH)^48^. Identification of cGMP as biomarker reflected its important role during pathogenic stress response as has been suggested^49^. Tyrosine was found to be up regulated biomarker in the results and tyrosine metabolism, like phenylalanine and tryptophan metabolism are interlinked with phenylpropanoid pathway which further relates to plant defense^50^. Pantothenic acid (vitamin B5) being the core of coenzyme A, is essential molecule in plant metabolism^51^ and its up-regulated accumulation as well as enrichment of pantothenate/CoA biosynthesis (Fig. 6a) interconnects with the plant responses against stress challenge. Vitamins like riboflavin and vitamin-derived metabolites act as activators of plant development and repressors of biotic /abiotic stresses due to their involvement in signaling pathways and tolerance^52^.

Pathway mapping using comprehensive KEGG database and PLANTCYC PMN integrated metabolite features with biological context to evaluate functions^53^. Analysis revealed 106 prominent KEGG metabolic pathways to which annotated metabolites were enriched. High enrichment of metabolite features in metabolic pathways (n = 530 hits) (Fig. 6) seems justified because of their role in the modulation of primary metabolic processes during pathogen interaction to support cellular energy requirements under biotic stresses for activating defense responses^54^. Primary metabolic pathways represent chain of reactions needed for photosynthesis, glycolysis, tricarboxylic acid cycle and synthesis of amino acid, lipids and fatty acids inside cells for sustaining cellular processes under stress^41^. Plant’s primary metabolism is compromised under stressed conditions to balance metabolic shifts from source to sink to regulate secondary metabolite production^55^ and this has been observed in metabolite enrichment results (Fig. 6). Metabolite features were enriched with high hits in secondary metabolite pathways (n = 416 hits) in tomato leaves, thereby indicating secondary metabolic reprogramming, which is supposed to initiate deterrence against disease and activate defense-related metabolic responses for self-protection^40,43^.

KEGG mapping also showed enriched biosynthetic pathways for the biosynthesis of cofactors, oxocarboxylic acids, steroids, carotenoids, vitamins, brassinosteroids, ubiquinone and terpenoids-quinones. Many of these pathways are interconnected with the metabolism of amino acids which are typical precursors of secondary metabolites such as phenolics and alkaloids^56^. Cofactors for which vitamins, pantothenic acid, folate, methionine, folic acid and pyridoxals are the precursors^57^, are essential for metabolic processes^58^ to regulate enzyme functions and thus determine plant growth. Oxocarboxylic acids play key role in the biology and metabolism of plants^59^ and acts for secondary metabolism^60^. Enriched brassinosteroid pathway provides the key stress responding plant growth promoting hormones^61,62^ in infected tomato plantsand further leads to the generation of other phytohormones such as ethylene involved in defense response^63^. Enrichment of carotenoid biosynthesis in the infected plants indicated plant strategies to employ such compounds which help in anti-oxidation and hormone synthesis during pathogen invasion^64,65^. Metabolites enriching ubiqinone pathway can function as electron transporters during photosynthesis^66^. Diterpenoid pathway offers direct defense functions^67^. Amino acid metabolism enrichment is crucial to affect metabolic process post pathogen interaction^68^. Overall, the enrichment analysis indicated high abundance of metabolite hits in different pathways and their dominance revealed modulation of plant responses towards defense, resistance and/or survival^69^.

Cellular overview of the pathway collage diagram in tomato leaves (Fig. 7) represented the involvement of up and down regulated discriminatory metabolites as shown in Tables 1 and 2 in different pathways. Such diagrams clearly reflect metabolic reactions and the pathways to which the annotated metabolites were involved, and thereby making a clearer understanding about their role in the pathway. Metabolite-metabolite interaction of up- and down regulated metabolites (Fig. 8) revealed linkages between these differential molecules post *A. solani* infection. With the help of network analysis, interactions among metabolites having contribution in plant responses against pathogen challenge were revealed to establish how similar metabolites within the same compound class are connected by biochemical reactions^70^.

Being sessile organisms, plants are continuously exposed to abiotic and biotic stress challenges in the environment. This has prompted for species-specific biosynthetic mechanisms that produce plethora of secondary metabolites, for which the precursors are usually derived from the primary metabolism. A shift from biosynthesizing primary compounds essential for growth and development to secondary metabolites that orchestrate defense architecture of the plants has been observed under biotic or abiotic challenges^71^. Looking into the prominent presence of putatively identified metabolites as discriminant biomolecules, key biosynthesis/metabolism pathways that were involved and critical biological role the metabolites and the pathways play in plants under pathogen-challenged conditions, they were considered as prospective biomarkers and crucial regulators of plant susceptibility regulating plant cellular metabolic responses against the early blight pathogen in tomato.

## Materials and methods

### Plant material and growth conditions

Early blight (*A. solani*) susceptible tomato (*S. lycopersicum* var. Kashi Aman) was grown in glass house conditions and inoculated with the pathogen^72^. Briefly, seeds were surface sterilized by using 2% sodium hypochlorite solution for 5 min and after drying, were seeded in earthen pots (20 × 20 × 14 cm) having 5 kg sterilized mixture of soil, cocopeat, perlite and vermiculite (2:1:1:1; w/w). After 3 weeks, seedlings were transplanted in 10 separate pots and allowed to grow in glass house conditions. Lower leaves were inoculated with the pathogen inoculum (2.1 × 10^4^ conidia mL^−1^) at early flowering stage (60 days) using sterilized bruise^73^. Pots were kept for disease development under 86–90% relative humidity. After 3 days of inoculation, upper leaves were collected from diseased and control (non-inoculated) plants for metabolome extraction. The disease incidence (%) was obtained at the time of sampling as reported earlier^72^. Plants (either cultivated or wild), including the collection of plant material, complied with relevant institutional, national, and international guidelines and legislations.

### Metabolite profiling: sample preparation, metabolites extraction and data acquisition using LC–MS/MS

For capturing comprehensive number of both polar and non-polar metabolites from plant leaf samples, 2 g of frozen leaf tissues were pulverized and extracted by sonication in triplicate with HPLC grade methanol (5 mL). Samples were centrifuged at 6000*g* (4 °C, 15 min) and dried on rotary evaporator (50 °C). Dried extracts from replicated samples were reconstituted in 1 mL HPLC-grade methanol and filtered through 0.22 µM syringe filter. Samples were pooled together and 200µL of the mixed and vortexed solution was transferred to capped dark glass vials at 4 °C. For each sample 3 biological replicates from two independent experiments were analyzed by LC–MS/MS. The analysis included quality samples comprising blanks and technical replicates as described by Mhlongo et al. (2021)^74^.

Leaf extract samples from pathogen-challenged and non-challenged plants were separated using Dionex Ultimate 3000 HPLC system 15µL per sample as injection volume was analyzed on Hypersil Gold C-18 (2.1 × 100 mm, 1.9 µm) column at 35 °C using mobile phase (A: 0.05% formic acid in water and B: 0.05% formic acid in acetonitrile) at a flow rate of 350 µl min^−1^ for 31 min. The elution conditions were initially 5% solvent B for 0 to 2 min, further increased from 5 up to 95% (solvent B) for 2.01–22.0 min, then 95% for 22.01–27.0 min and finally 5% for 27.01–31.0 min.

Data acquisition on full scan MS range (100–1500 *m/z*) was obtained in negative ionization mode by Q Exactive mass spectrometer (Thermo Scientific, USA) coupled to HPLC system at the first order resolution. The ion source parameters were sheath gas flow rate 60 au (arbitrary unit), aux gas flow rate 20au, sweep gas flow rate 10 au, capillary voltage (+) 3.2 kV and capillary temperature 275 °C. The S-Lens level was kept at 55 rf and probe and aux gas heat temperatures remained at 250 and 350 °C, respectively. Sample run was performed in three technical replicates along with quality control blank samples.

Tandem mass spectrometry (MS/MS) parameters improve mass fragmentation patterns. For MS/MS, scan range was 100–2000 *m/z*, isolation offset *m/z* 0.5, collision energy (CE) 25-45 eV, maximum IT 50 ms, loop count 5, MSX count 1, resolution 35,000, microscans 1 and isolation window *m/z* 2.0. Further, Data Dependent Acquisition (DDA) and automated precursor ion selection methods were described by Singh et al. (2023)^75^. For MS^2^ spectra, two blanks and three quality checks (QCs) were ensured during system conditioning.

### Data analysis and metabolite annotation

The raw data generated from LC–MS/MS was subjected to Compound Discoverer 3.3 (Thermo Scientific, USA) for data analysis as per the parameters described previously^75^. The raw files were pre-processed and analyzed to yield peak intensity, retention time (*rt*), abundance, missing values and adjoint ion combinations after appropriate normalization of metabolite features (*m/z*). During analysis, data normalization was performed with the combined preset parameters grouped into three categories in MetaboAnalyst 5.0 (https://www.metaboanalyst.ca) by selecting median, log transformation (base10) and Pareto scaling. The overall annotation of metabolite features was performed by Functional Analysis module using Pathway Enrichment and Network Analysis sub-modules. Statistical (one factor) and chemometric analysis was performed to establish distinction within the groups and identify discriminant metabolite biomarkers.

Functional annotation of the metabolite features was obtained from MetaboAnalyst 5.0 (Functional Analysis module) which required *m/z*, *p* value, *t* score and *rt* as input file and generated annotated compounds having *m/z*, *rt*, KEGG identifiers (ids) of the matched metabolites and ion forms based on *Arabidopsis thaliana* plant database. The KEGG ids when submitted to PLANTCYC PMN using *S. lycopersicum* database (http://plantcyc.org) resulted in the annotation of the metabolites as per the Metabolomics Standards Initiative (PI-level 2 annotation)^76^ reporting minimum standards for chemical analysis^77,78^. Pathway mapping was obtained using KEGG Mapper. Putatively annotated biomarker metabolites were validated for their robustness using Multivariate Exploratory Receiver Operating Characteristic (ROC) Curve analysis that was based on three multivariate algorithms, support vector machines (SVM), partial least squares discriminant analysis (PLS-DA) and random forests in MetaboAnalyst 5.0. Pathway Collage, a diagram containing user-specified set of pathways of the annotated putative metabolites was prepared using PMN (Pathway Collages (plantcyc.org) while metabolite-metabolite interaction was performed by MetaboAnalyst 5.0.

### Statistical analysis

Univariate and multivariate data analysis was performed by Statistical Analysis (one factor) module of MetaboAnalyst 5.0. By the means of Debiased Sparse Partial Correlation (DSPC) network^79^, which comprised nodes as input metabolites and edges as the association measures, correlation network was developed. For other purposes, SPSS 16.0 was applied for one-way ANOVA (analysis of variance) using Student t-test.

## Conclusion

The study explored metabolomic responses of susceptible tomato variety Kashi Aman challenged with early blight pathogen. Out of 8487 infection-exclusive and 8742 non-infection exclusive metabolite features, 481 significantly up- regulated and 548 down regulated features were identified. OPLS-DA model based VIP score ≥ 1.0 coupled with FC threshold ≥ 2.0 suggested 43 up regulated and 23 down regulated annotated discriminant metabolites which belonged to different group of compounds arising from various metabolic pathways. Pathway mapping revealed that the annotated features with KEGG ids were distributed across 106 biosynthetic pathways of primary and secondary metabolism. Secondary metabolism pathways predominantly included secondary metabolites (other antibiotics), steroids, brassinosteroids, mono-, sesqui-, di- and triterpenoids, carotenoids and ubiquinone/other terpenoids. Network impact analysis indicated dominance of those pathways which aid to modulate plant biological responses towards defense, resistance, and/or survival. The ROC-based annotated biomarker metabolites apigenin-7-glucoside, uridine, homocyctine, adenosyl-homocysteine, cGMP, tyrosine, pantothenic acid, adenosine, riboflavin and azmaline could be considered as potential diagnostic biomarker biomolecules in tomato leaves in response to pathogen challenge. Deciphering pathogen-driven key metabolic changes in tomato leaf tissues, altered metabolic pathways influencing plant responses to pathogen and potential biomarkers in challenged plants using metabolomics could help in designing diagnostic tools. The identified strong discriminant metabolites may help in developing metabolite-QTLs (mQTLs) having implications in molecular breeding for resistance in crop plants against biotic stresses. Results from the pathway mapping may also be implicated for targeted modifications in key pathways through biotechnological tools including genome editing coupled with the classical breeding for improving crop performance against pathogens in the field.

### Supplementary Information

Supplementary Figure S1.

Supplementary Figure S2.

## Data Availability

The datasets used and/or analysed during the current study available from the corresponding author on reasonable request.
