# Supplementary figures and images for: Metabolomics of early blight (Alternaria solani) susceptible tomato (Solanum lycopersicum) unfolds key biomarker metabolites and involved metabolic pathways
Source: Sci Rep. 2023 Nov 29;13:21023. doi: 10.1038/s41598-023-48269-0 (PMC10687106; doi:10.1038/s41598-023-48269-0)

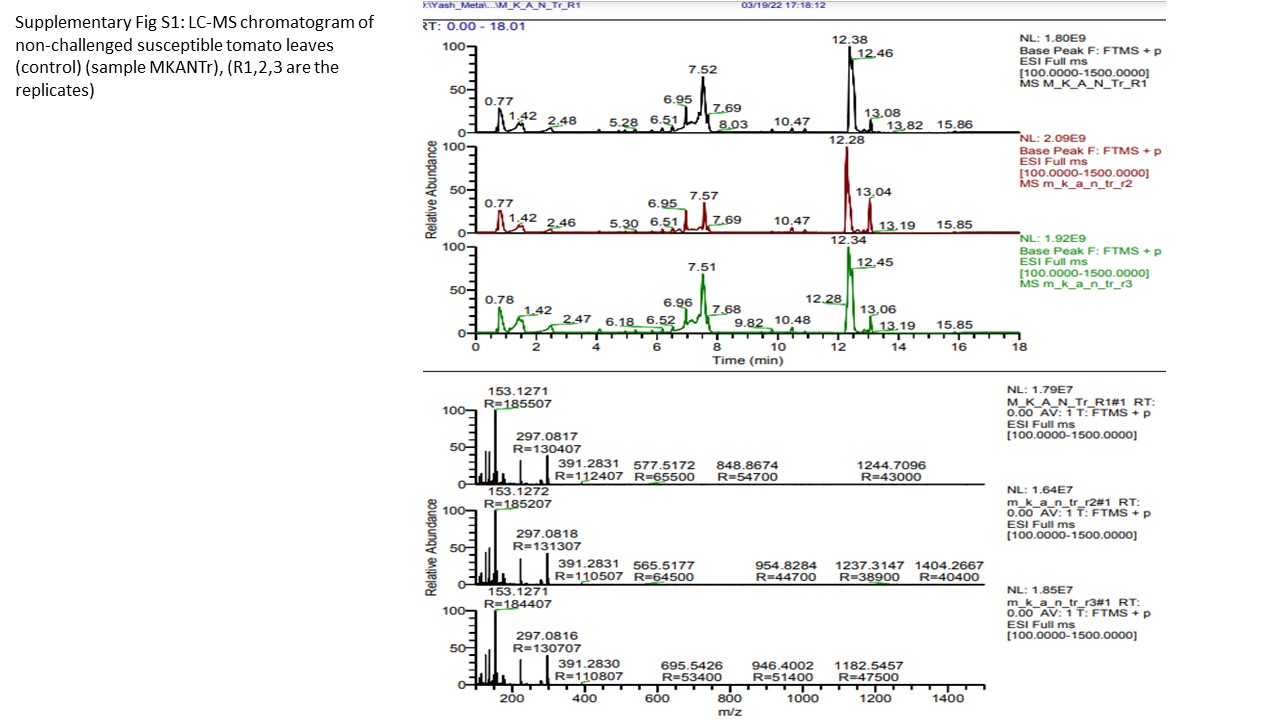

Supplement: Supplementary file 1 — Supplementary Figure S1. [file 41598_2023_48269_MOESM1_ESM.jpg]

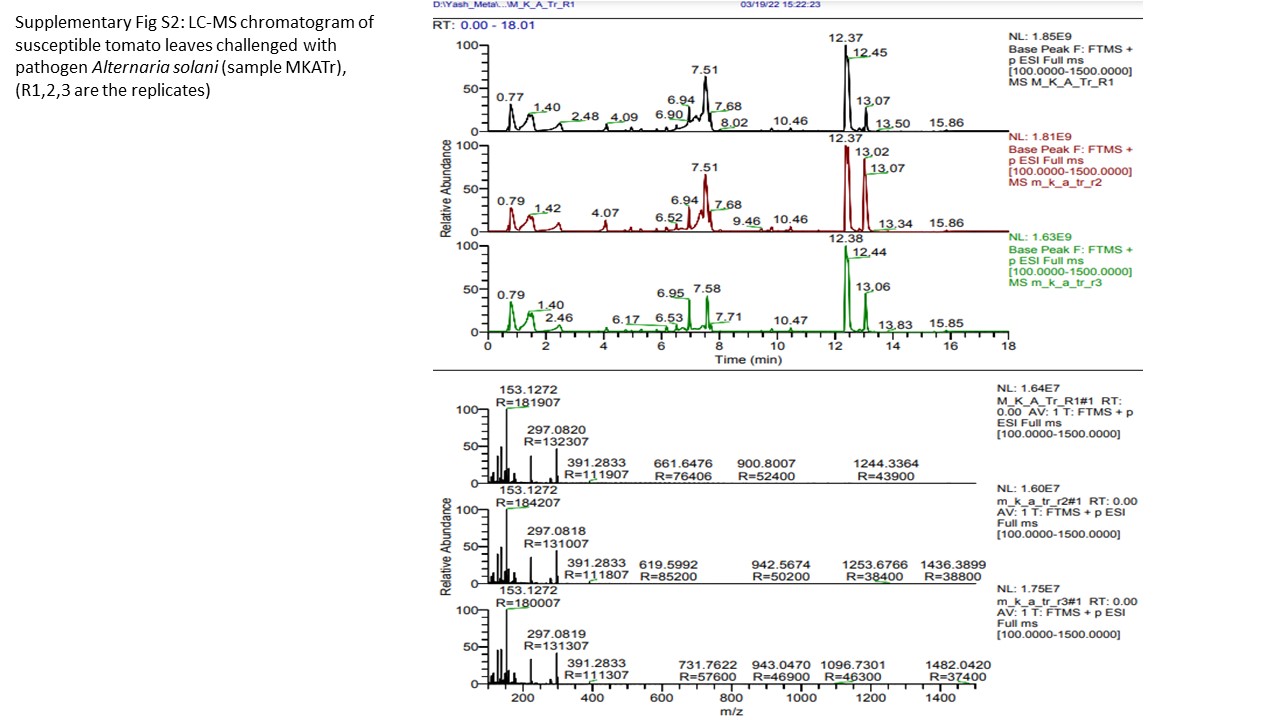

Supplement: Supplementary file 2 — Supplementary Figure S2. [file 41598_2023_48269_MOESM2_ESM.jpg]
